# Enhanced production of polysaccharides and triterpenoids in *Ganoderma lucidum* fruit bodies on induction with signal transduction during the fruiting stage

**DOI:** 10.1371/journal.pone.0196287

**Published:** 2018-04-25

**Authors:** Liyun Ye, Shengrong Liu, Fan Xie, Lili Zhao, Xiaoping Wu

**Affiliations:** 1 Mycological Research Center, Fujian Agricultural and Forestry University, Fuzhou, Fujian, China; 2 College of Life Science, Ningde Normal University, Ningde, Fujian, China; Leibniz-Institut fur Naturstoff-Forschung und Infektionsbiologie eV Hans-Knoll-Institut, GERMANY

## Abstract

*Ganoderma lucidum* is a medicinal mushroom that has been widely used in East Asia for the treatment of various diseases. The pharmacological activity of this fungus is primarily attributable to the polysaccharides and triterpenoids. In this study, to obtain the fruit bodies with improved content of active constituents, we examined the effect of salicylic acid (SA) and calcium ion on the biosynthesis of polysaccharides and triterpenoids by spraying the chemicals during the fruiting. To explore the underlying mechanisms for the variation, the transcripts of related genes involved in the polysaccharide and triterpenoid biosynthesis were measured. Results showed that Ca^2+^ had no effect on production of polysaccharides and triterpenoids, whereas SA increased triterpenoid content by 23.32%, compared to the control, but it had little influence on polysaccharide production. Interestingly, the combined induction increased polysaccharide and triterpenoid content by 9.02% and 13.61%, respectively, compared to the control. Under Ca^2+^ induction, the transcript of *ugp* gene in the polysaccharide biosynthetic pathway up-regulated in all three stages (mycelium, primordium, and fruit body), while *pgm* and *gls* gave no response in the mycelium and primordium stages, and up-regulated in the fruit body stage. Differently, six key triterpenoid biosynthetic genes including *hmgr*, *hmgs*, *mvd*, *fps*, *sqs*, and *ls* did not respond to the induction. In the case of SA and combined induction, *pgm* and *ugp* were up-regulated in all three stages, while *gls* showed an increased expression in the primordium stage and no response in other stages. The six triterpenoid biosynthetic genes were up-regulated in all three stages. The present study provides a useful approach to producing *G*. *lucidum* fruit bodies with high polysaccharide and triterpenoid content. This is important to the *G*. *lucidum* industry.

## Introduction

*Ganoderma lucidum* (Fr.) Karst, commonly known as Lingzhi in China and Reishi in Japanese, is a basidiomycete fungus belonging to the Polyporaceae family. It is widely used in East Asia as a remedy for minor health disorders and to promote vitality and longevity. As a traditional Chinese medicine, it has been long used to treat many diseases such as hepatitis, hypertension, arthritis, bronchitis, nephritis, hypercholesterolemia, and cancer in oriental countries [1–3]. Recent studies on *G*. *lucidum* have shown that it has potent biological activities, including anticancer, antioxidant, and immunomodulating effects [4,5]. Numerous studies of *G*. *lucidum* revealed that polysaccharides and triterpenoids are the two major active molecules responsible for most of the pharmcological activities of *G*. *lucidum*. To gain insights into the mechanisms of polysaccharides and triterpenoids for medicinal benefits, their effects on various human cell lines have been investigated extensively [6–8].

Owing to its health-promoting benefits and therapeutic actions, there is a rapidly growing demand for *G*. *lucidum* in the market. Currently, to obtain large quantities of fruit bodies for commercial use in the preparation of health food and medicines, artificial cultivation of *G*. *lucidum* is widely employed in China, Japan, and Korea at a commercial scale. As the consumption of *G*. *lucidum* largely depends on its healthy and medicinal benefits, which, in turn, depend on the active components, namely, polysaccharides and triterpenoids, their content in the fruit bodies has become a major concern for researchers, cultivators, and consumers. However, it is reported that the content of polysaccharides and triterpenoids within *G*. *lucidum* fruiting bodies was very low, often around 0.5% [9] and 1.0% [10], respectively; this limits the health-promoting benefits and therapeutic efficiency of this fungus.

**Fig 1:**
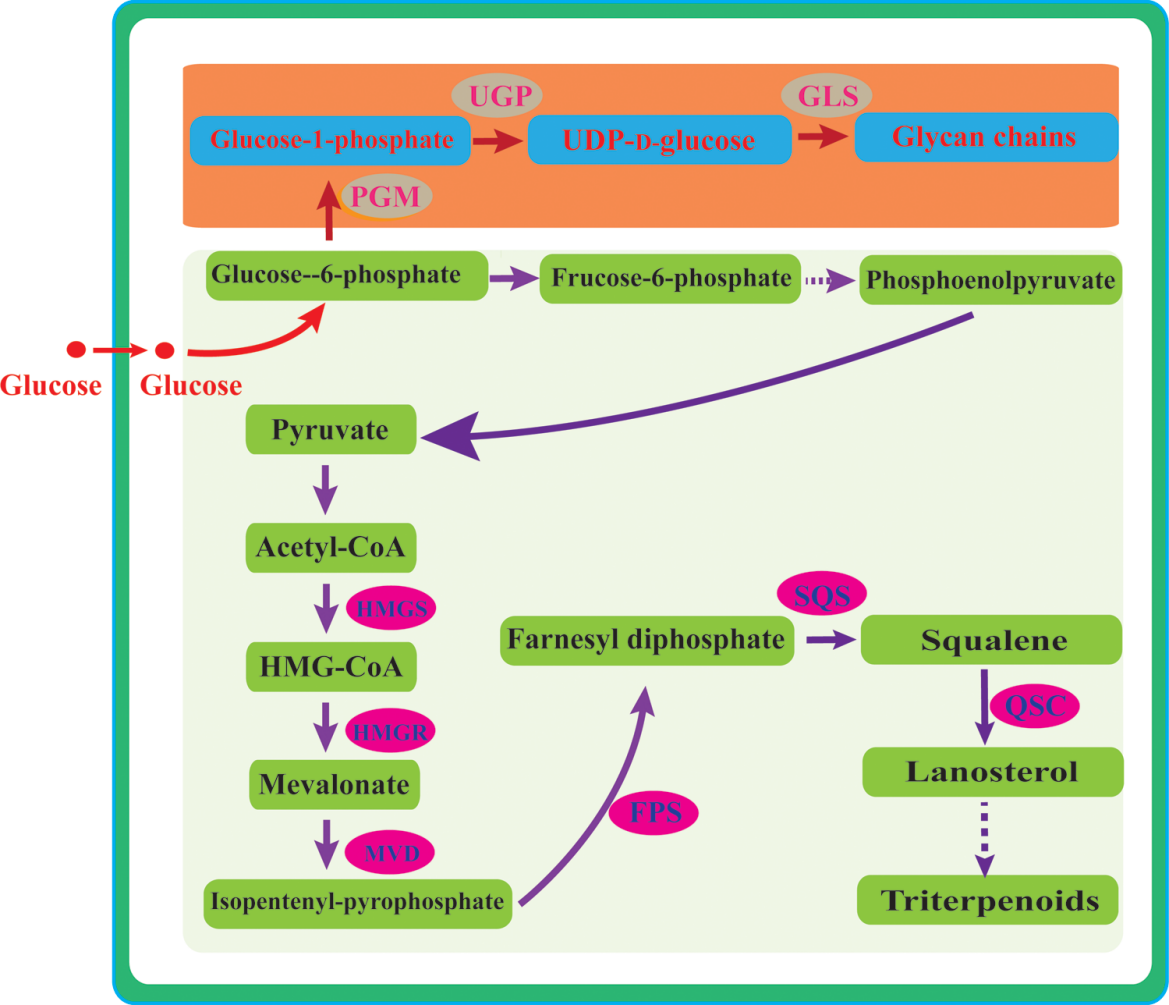
Biosynthetic pathways of polysaccharides and triterpenoids in *G*. *lucidum*. Dashed lines indicate steps consisting of multiple enzyme-catalyzed reactions. Symbols: HMG-CoA, hydroxy-3-methylglutaryl-Coenzyme A; HMGS, HMG-CoA synthase; HMGR, HMG-CoA reductase; MVD, mevalonate-5-pyrophosphate decarboxylase; IPP, isopentenyl-pyrophosphate; FPS, farnesyl pyrophosphate synthase; FPP, farnesyl diphosphate; SQS, squalene synthase; and LS, lanosterol synthase.

Generally, the polysaccharide biosynthetic pathway involves the biosynthesis of nucleotide sugar precursors, assembly of the repeating monosaccharide units, and polymerization [11–14] (Fig 1). Among the polysaccharide biosynthetic enzymes, phosphoglucomutase (PGM) and UDP-glucose pyrophosphorylase (UGP) are important enzymes in the biosynthesis of nucleotide sugar precursors. PGM catalyzes the conversion of glucose-6-phosphate into glucose-1-phosphate, while UGP catalyzes the reversible conversion of glucose-1-phosphate and UTP into UDP-d-glucose. β-1,3-Glucan synthase (GLS) is an enzyme related to polysaccharide synthesis; it catalyzes the repetitive addition of glucose units from UDP-glucose to the growing glycan chain [11,12,14]. However, the detailed steps and genes involved in polysaccharide biosynthesis are still poorly understood.

In *G*. *lucidum*, triterpenoids are synthesized via the mevalonate pathway (MVA) wherein acetyl-CoA is converted to 3-hydroxy-3-methylglutaryl-CoA (HMG-CoA) through a series of chemical reactions, and further to mevalonate (MVA) to isopentenyl-pyrophosphate (IPP) to farnesyl diphosphate (FPP) to squalene, and finally to lanosterol [15,16] (Fig 1). The steps leading to the production of triterpenoids from lanosterol are yet unknown, but, most likely, include a series of oxidation, reduction, and acylation reactions. Recently, a variety of genes involved in the MVA pathway have been cloned and characterized in *G*. *lucidum* [17–19]. 3-Hydroxy-3-methylglutaryl coenzyme A reductase (HMGR), squalene synthase (SQS), and lanosterol synthase (LS) were identified as key enzymes involved in triterpenoid biosynthesis, and their enhanced expression was found to promote the production of ganoderic acids, a kind of triterpenoids [20–22].

As the pharmcological activities of *Ganoderma* polycaccharides and triterpenoids have been gained wide acceptance in recent years, there has been an increased demand for them. Thus, efficient methods for the production of polycaccharides and triterpenoids are needed to be developed. As submerged culture has a great potential for higher mycelial production in a more compact space and shorter time, with few chances of contamination [23], this is viewed as a promising alternative for the production of *Ganoderma* polysaccharides, and triterpenoids especially ganoderic acids. Hence, many attempts such as optimization of fermentation conditions [24,25], development of new bioprocessing strategies [26] as well as overexpression of key biosynthetic genes in polysaccharide and triterpenoid biosynthesis pathways have been made to increase their production [27–28]. Most recently, the use of inducers like Cu^2+^ [29], acetic acid [30], methyl jasmonate [15], and phenobarbital [20] to increase the active components in *G*. *lucidum* submerged fermentation has drawn great interest, and offered promising results. In our previous study, a *G*. *lucidum* hybrid with relatively high polysaccharide and triterpenoid content in the fruiting body was bred [31]. However, although artificial cultivation is still the major method used for production of *G*. *lucidum* fruiting bodies, no report dealing with the production of fruiting bodies with high content of active components using signal transduction induction is available.

Salicylic acid (SA) is a signaling molecule in plant cells with multiple effects on plant growth, development, and secondary metabolism, which has been widely used as an inducer to promote the synthesis of secondary metabolites [32], and it has been reported that this inducer can promote ganoderic acid biosynthesis considerably during the submerged culture of *G*. *lucidum* [33,34]. On the other hand, Ca^2+^ is thought to be closely related with hyphal extension, branching, and differentiation in higher fungi, including mushroom [35,36], and it is reported that Ca^2+^ is involved in calcineurin signal transduction in *G*. *lucidum*, and its addition significantly improved the production of ganoderic acids [37]. Later, the culture strategy of Ca^2+^ addition combined with nitrogen limitation was developed, and significant improvement of content of ganoderic acid T in the cultured mycelia was achieved [22].

The present study aimed to improve the content of polysaccharides and triterpenoids in the fruiting bodies of *G*. *lucidum* during artificial cultivation using signal transduction induction, with SA and Ca^2+^ as the inducers. To explore the underlying mechanisms responsible for the enhanced content, the transcripts of genes encoding key enzymes in the polysaccharide and triterpenoid biosynthetic pathways were assayed. This research demonstrates that the combined use of SA and Ca^2+^ for induction can significantly improve content of polysaccahrides and triterpenoids. The newly developed method holds great promise for application in the production of *G*. *lucidum* fruiting bodies with improved polysaccharide and triterpenoid content. The present study also helps to better understand the regulation of triterpenoid biosynthesis in *G*. *lucidum*.

## Materials and methods

### Fungal strains

*G*. *lucidum* strain J-7/AL-2, a hybrid obtained in our lab and stored in the Mycological Research Center of Fujian Agriculture and Forestry University, was used in this study. It was maintained on potato dextrose agar slants and sub-cultured at regular intervals of 2 months.

### Preparation of spawn

Spawn was prepared in 750-mL glass bottles. Sawdust (78%) was supplemented with 20% wheat bran and 2% gypsum based on dry weight, and adjusted to a moisture content of 65%. The moistened substrate (500 g) was filled in each bottle, which was closed with cotton plugs. The bottles were sterilized at 121°C for 2 h, and cooled to room temperature. Then, an actively growing mycelium agar plug (10 mm in diameter) was inoculated on the surface of the sterilized substrate. The inoculated substrate was incubated at 25°C and 60–70% relative humidity for mycelial colonization, and subsequently used as spawn after the substrate was completely colonized by the mycelium.

### Substrate preparation, inoculation and spawn running

A substrate (75% sawdust, 20% wheat bran, 3% corn flour, 1% gypsum, and 1% lime, based on the dry weight of ingredients) was used. The moisture content was adjusted to 65%. The mixed substrate (1000 g) was transferred into a polypropylene bag (18 cm in width × 36 cm in length), and packed. Then, a single, vertical hole (2 cm in diameter and 18 cm in depth) was made in the center of the packed substrate for inoculation and aeration. The opening of the bags was closed with cotton plugs using a plastic ring. The bags were autoclaved at 121°C for 2 h. After cooling, spawn at 5% (w/w) ratio was inoculated into the holes and uniformly distributed onto the surface of the substrates. Spawn running was at 25°C and 60–70% relative humidity in the dark, and aeration was performed once daily. After the substrate was completely colonized by mycelium, the bags were further incubated for 7 days for the mycelium to mature.

### Fruiting and signal induction

Bags containing matured mycelium were initially cut open at the upper portion, and transferred to a fruiting room at 25°C, 85–95% relative humidity, and 500 lux light density. Ventilation was performed twice daily. The bags were subjected to treatment by spraying SA (10 mg/mL in ethanol) and calcium chloride (30 mg/mL in distilled water), alone or in combination on opening mouths at regular intervals of 3 days for a total period of 30 days. For Ca^2+^ induction, at each instance, 16 mL of prepared calcium chloride solution was sprayed on each bag. The bag treated with distilled water was used as the control. For SA induction, 20 mL of the SA ethanol solution was sprayed on each bag at a time, and the ethanol bag was set as the control. For combined induction, both solutions were used at the same concentration and volume as those used in their respective individual inductions. The bags treated with distilled water and ethanol were used as the control.

### Harvesting and determination of biological efficiency

When the white margins of the pileus disappeared, mature fruiting bodies were collected from the bags, weighed, and dried at 60°C to a constant weight. Mushroom yield was recorded only for the first flush as the dry weight. Biological efficiency was determined as the ratio of the weight of freshly harvested mushrooms to the dry weight of the substrate; this was expressed as a percentage.

### Quantification of polysaccharides

The dried fruit bodies were sliced and ground into a fine powder (60 mesh). This powder (2 g) was suspended in 95% ethanol at room temperature for 48 h with stirring to remove some colored materials, monosaccharide, oligosaccharides, and small molecule materials [38]. The cooled extract was discarded and the residue was washed with 95% ethanol. Then, the residue was extracted with 40 mL of double-distilled water for 3 h at 90°C and filtration. The extraction procedure was carried out twice. The combined aqueous extracts were concentrated in a rotary evaporator under reduced pressure at 50°C and filtered. The filtrate was precipitated with 4 times volume of 95% ethanol at 4°C overnight to obtain crude polysaccharides. The precipitated polysaccharides were pelleted by centrifugation at 8000 ×g for 10 min at 4°C and the supernatant was discarded. The pellet was then re-suspended in an equal volume of 75% ethanol to remove oligosaccharides [39] and centrifuged against as above. Then, the precipitate was dried at 50°C. Total polysaccharide content was measured by the phenol-sulfuric acid method with glucose as the standard [40].

### Quantification of triterpenoids

Two grams of the fine powder of whole dried fruit body was mixed with 50 mL of 95% ethanol at room temperature for 2 h. Next, the mixture was ultrasonicated at a frequency of 20 kHz in an ultrasonic bath (KQ-3200DE; Kunshan Ultrasonic Instrument Co., Ltd., China) for 30 min. The supernatant was obtained by centrifugation at 5000 ×*g* for 10 min. The residual powder was re-extracted by the same method. The two supernatants were combined. The extracted solution was mixed and filtered through analytical filter paper, and then, evaporated to dryness at 35°C in vacuum. The dry extract was dissolved in 5 mL of methanol and filtered through a 0.45-μm membrane filter unit. Total triterpenoid content was determined based on a coloring reaction involving vanillin-perchloric acid and glacial acetic acid with triterpenoids, as described by Chen et al. [10], with ursolic acid as the reference.

### Chromatography

To detect the possible post-induction changes in the levels of individual ganoderic acids in the fruit body, the methanol extract was used to perform the assay on an Agilent series 1100 HPLC instrument (Agilent, USA) equipped with a quaternary pump, a diode-array detector (DAD), an autosampler, and a column compartment. The sample (10 μL) was separated on a Zorbax SB-C_18_ column (5 μm, 4.6 × 250 mm; Agilent). The mobile phase consisted of acetonitrile (CH_3_CN) and water containing 0.1% (v/v) CH_3_COOH, with a gradient from 30% to 32% CH_3_CN over the first 40 min, then up to 40% in 20 min, and held at 40% CH_3_CN for another 5 min. The flow rate was 1.0 mL/min, and column temperature was set at 35°C. The DAD detector was monitored at 252 nm. The mixture of ganoderic acids A, C, C2, E, F, and lucidenic acid A in methanol was used as the reference standard.

### RNA isolation and cDNA synthesis

To analyze gene expression at different stages of fruit body development of *G*. *lucidum* with or without induction, the mycelium was isolated from the mycelium, primordium, and fruit body stages in solid cultures. The isolation for each stage was performed at the same time for all different inductions and controls. For the mycelium stage, the mycelia-colonized substrate on the surface of treated bags was sampled. As for the fruit body stage, mycelial tissue was isolated from the white margin of pileus of *G*. *lucidum*. All samples were frozen in liquid nitrogen, and ground to fine powder with a mortar and pestle. Total RNA in *G*. *lucidum* was extracted using an Omega plant RNA extraction kit according to the manufacturer’s instruction. RNA concentration was determined using Nanodrop 2000 (Thermo). The total RNA obtained was treated with RNase-free DNase I (Invitrogen) to remove genomic DNA. Then, 1 μg of total RNA was reverse-transcribed using the Superscript RNase H-First-strand synthesis kit (Invitrogen), following the manufacturer’s recommendations.

### Real-time quantitative PCR (qRT-PCR)

The transcript levels of *hmgs*, *hmgr*, *mvd*, *fps*, *sqs*, and *ls* genes and *pgm*, *ugp*, and *gls* genes involved in the triterpenoid and polysaccharide biosynthetic pathways, respectively, were detected by qRT-PCR. For qRT-PCR assay, the cDNA was quantified using a Mastercycler ep realplex 2S detection system (Eppendorf). PCR was performed according to the procedure provided by the manufacturer of Maxima SYBR Green qPCR Master Mix (Fermentas). After denaturation at 95°C for 10 min, amplification occurred in three steps: 15 s of denaturation at 95°C, 30 s of annealing at 55°C, and 30 s of extension at 72°C, for a total of 40 cycles. Transcript levels were determined using the standard curve method and normalized against the 18S rDNA gene (an internal control). For each gene, the expression in all three developmental stages (mycelium, primordium, and fruit body) in the control was defined as 1.0, and the results of the induced samples were expressed as the ratio relative to that of the control under the same developmental stages. Primers used are listed in Table 1.

**Table 1:** Primer pairs used for real-time quantitative PCR in this study.

Target genes	Primers	Sequences	References
*hmgs*	Forward	5'-CCCATCAACGCTTCCACCA-3'	15
	Reverse	5'-GCTCCTCCTCCGAAATGC-3'	
*hmgr*	Forward	5'-TCGCAGTGGCACAGGAGC -3'	21
	Reverse	5'-CCCGGTGTTGGTGTTAGAAG -3'	
*mvd*	Forward	5'-TCGGACTCGCTTGCGGTAGA-3'	15
	Reverse	5'-CGTGCTTGATACGGTGCTG-3'	
*fps*	Forward	5'-CCTCATCACCGCTCCAGAA-3'	15
	Reverse	5'-AGGGCGACGGGAAGGTAGAA-3'	
*sqs*	Forward	5'-TGACTCTTCCTGACGAGA-3'	21
	Reverse	5'-GTGGCAGTAGAGGTTGTA-3'	
*ls*	Forward	5'-CTTCCGCAAGCACTACCCG-3'	21
	Reverse	5'-AGCAGATGCCCCACGAGCC-3'	
*18S rRNA*	Forward	5'-TATCGAGTTCTGACTGGGTTGT-3'	21
	Reverse	5'-ATCCGTTGCTGAAAGTTGTAT-3'	
*pgm*	Forward	5'-GGGCCTGAGGAAGAGGGTGA-3'	27
	Reverse	5'-CGGTTTCGGGGGAGAAGTAG-3'	
*ugp*	Forward	5'-TGGTCTCGGAACTTCTATGGG-3'	27
	Reverse	5'-CAGTGCTTCTTCTCGTCCTCA-3'	
*gls*	Forward	5'-TCGTTTGGGTTGGGTCTGT-3'	27
	Reverse	5'-GAAGCCCTTGTCGCTCTGC-3'	

### Experimental design and statistical analysis

The fruiting test was performed with a completely randomized design including 30 replicates. All data are expressed as the mean ± standard deviation (SD). Data were subjected to analysis of variance using the Student’s t-test, and the mean values indicating statistical significance were compared by Duncan’s multiple-range test using the SPSS 17.0 software (SPSS Inc., Chicago, IL, USA). Differences were considered significant at *p*<0.05.

## Results

### Fruiting parameters and polysaccharide and triterpenoid content in the fruiting bodies of *G*. *lucidum* in response to different inductions

As show in Table 2, the time from inoculation to primordium formation and first harvest was unaffected by the inducers, without any statistical difference (*p*>0.05) in mushroom yield and biological efficiency also. There was no statistical difference in the content of both polysaccharides and triterpenoids in *G*. *lucidum* fruiting bodies between the Ca^2+^ induction and the control. As to SA treatment, compared to the control (12.18 mg/g dry weight), triterpenoid production significantly improved, reaching a dry weight of 15.02 mg/g, with an increase of 23.32%, but there was little effect on polysaccharide production. In the case of combined induction, the content of polysaccharides and triterpenoids was improved, from 12.41 to 13.53 mg/g and 12.64 to 14.36 mg/g, respectively, but the improvement in polysaccharide production was less than that observed in triterpenoid production.

**Table 2:** Days to primordium formation and first harvest, mushroom yield, biological efficiency, and polysaccharide and triterpenoid content in the *G*. *lucidum* fruiting bodies induced by different treatments.

Treatment	Days to primordium formation	Days to first harvest	^a^Mushroom yield (g/bag)	Biological efficiency (%)	^b^Polysaccharide content (mg/g)	^b^Triterpenoid content (mg/g)
Ca^2+^	34.31 ± 2.03^**a**^	53.22 ± 2.23^**a**^	57.41 ± 4.26^**a**^	12.26 ± 0.31^**a**^	12.51 ± 0.26^**a**^	11.47 ± 0.22^**a**^
SA	35.04 ± 1.46^**a**^	54.78 ± 1.47^**a**^	54.75 ± 2.71^**a**^	11.94 ± 0.28^**a**^	12.62 ± 0.31^**a**^	15.02 ± 0.17^**a**^^**b**^
Ca^2+^ + SA	35.36 ± 1.26^**a**^	52.81 ± 3.62^**a**^	56.53 ± 4.18^**a**^	12.17 ± 0.34^**a**^	13.53 ± 0.21^**b**^	14.36 ± 0.25^**b**^
Water (control)	35.44 ± 3.12^**a**^	54.41 ± 1.26^**a**^	54.36 ± 3.76^**a**^	11.73 ± 0.64^**a**^	12.68 ± 0.19^**a**^	12.03 ± 0.38^**a**^
Ethanol (control)	36.13 ± 1.87^**a**^	52.27 ± 2.12^**a**^	55.24 ± 3.07^**a**^	12.06 ± 0.83^**a**^	12.35 ± 0.11^**a**^	12.18 ± 0.14^**a**^
Water + Ethanol (control)	35.28 ± 1.20^**a**^	53.73 ± 2.71^**a**^	56.07 ± 2.23^**a**^	12.31 ± 0.52^**a**^	12.41 ± 0.32^**a**^	12.64 ± 0.11^**a**^

For polysaccharide and triterpenoid content: Values are the mean ± standard deviation of three independent bag samples. For all other parameters: Values are the mean ± standard deviation of 30 independent bag samples. Mean values in each column followed by the same superscript are not significantly different (*p*>0.05).

^a^based on wet weight.

^b^based on dry weight.

### Changes in the ganoderic acid pattern of the fruiting bodies in response to different inductions

Data on the content of several individual ganoderic acids are listed in Table 3. Compared to the control, the content of ganoderic acids A, C, C2 and E increased, with the most enhancement by 87.42% on ganoderic acid E, and that of ganoderic F kept almost unchanged, but that of lucidenic acid A decreased after calcium ion induction. As for SA treatment, the contents of ganoderic acids A, C, C2 and F increased considerably by 48.38%, 60.22%, 248.15% and 13.07%, respectively, whereas a decrease was observed in the contents of ganoderic acid E and lucidenic acid A. In the case of combined induction, an increase was observed in the contents of ganoderic acids A, C, C2, and F, in the range of 14.06–329.17%, but a decrease was observed in the contents of ganoderic acid E and lucidenic acid A. The highest total content of the detected individual ganoderic acids was 15.98 μg/mg dried fruiting body achieved by the combined induction, followed by single SA induction at 15.80 μg/mg, and Ca^2+^ induction at 14.72 μg/mg. The three controls gave the least content value of around 12.81 μg/mg. S1 Fig shows the HPLC chromatograms of extracts of the fruit bodies after different treatments.

**Table 3:** Content of individual ganoderic acids in *G*. *lucidum* fruiting body, after exposure to different inductions.

Treatment	Ganoderic acid (μg/mg dried fruit body)					
	A	C	C2	E	F	Lucidenic acid A
Ca^2+^	1.52 ± 0.06^a^	5.13 ± 0.21^ab^	0.36 ± 0.03^a^	2.83 ± 0.13^a^	3.24 ± 0.18^a^	1.64 ± 0.08^ac^
SA	1.84 ± 0.14^b^	7.21 ± 0.41^cd^	0.94 ± 0.06^b^	0.53 ± 0.06^b^	3.72± 0.09^ac^	1.56 ±0.07^b^
Ca^2+^+ SA	1.46 ± 0.09^a^	6.51 ± 0.34^bc^	1.03 ± 0.14^b^	1.08 ± 0.08^c^	4.37 ± 0.12^b^	1.53 ± 0.05^b^
Water (control)	1.29 ± 0.12^c^	4.67 ± 0.18^a^	0.26 ± 0.03^c^	1.51 ± 0.16^d^	3.39 ± 0.20^c^	1.72 ± 0.09^a^
Ethanol (control)	1.24 ± 0.09^c^	4.50 ± 0.13^a^	0.27 ± 0.02^c^	1.53 ± 0.09^d^	3.29 ± 0.16^a^	1.66 ± 0.11^ac^
Ethanol + water (control)	1.28 ± 0.12^c^	4.56 ± 0.20^a^	0.24 ± 0.03^c^	1.48 ± 0.13^d^	3.34 ± 0.11^c^	1.74 ± 0.08^a^

Values are the mean ± standard deviation of three independent samples; mean values in each column followed by the same superscript are not significantly different (*p*>0.05).

### Expression of three related genes in polysaccharide biosynthetic pathway in response to different inductions

**Fig 2:**
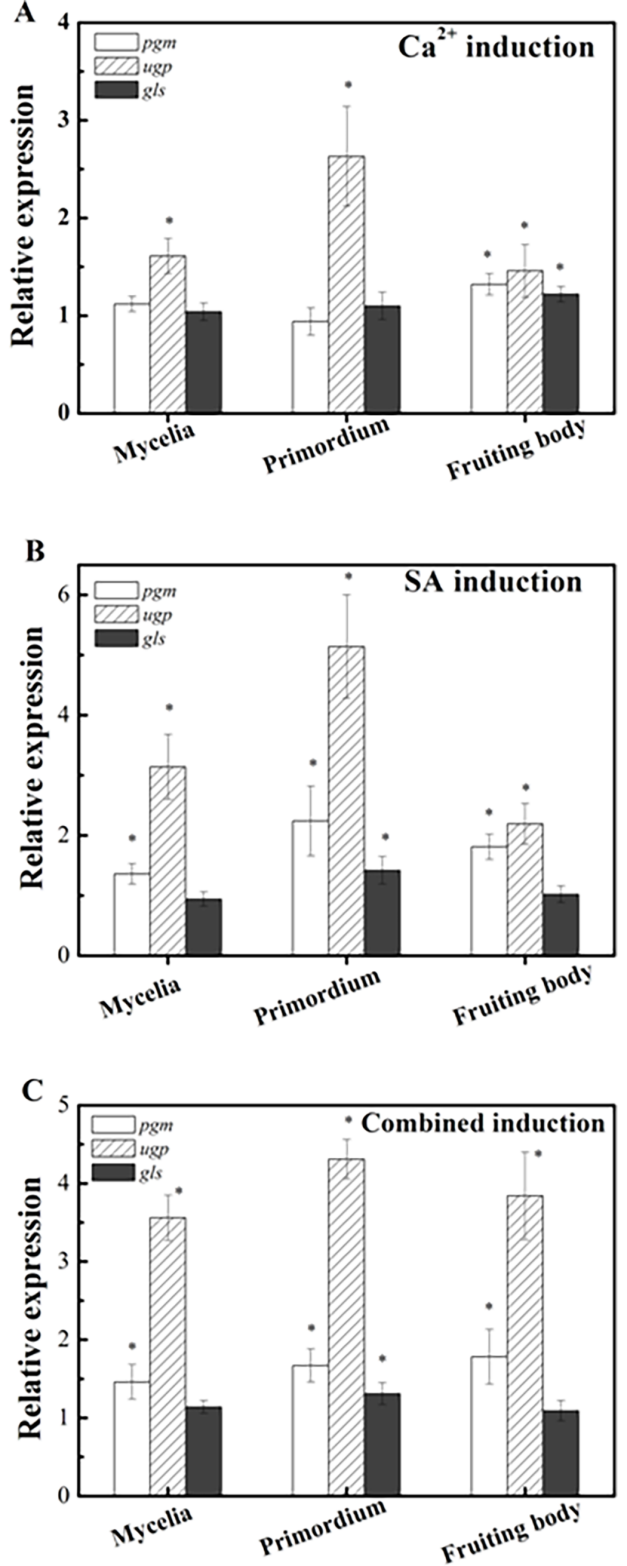
Transcript levels of *pgm*, *ugp*, and *gls* genes in the polysaccharide biosynthetic pathway during three stages of fruit body development of *G*. *lucidum* induced with Ca^2+^ and SA, alone or in combination. The measurement was carried out using samples after 3 days, 9 days, and 21 days of induction corresponding the mycelium, primordium, and fruit body stages, respectively. Expression of the control samples in all three developmental stages is defined as 1.0, and expression levels of the induced samples are displayed as the ratio relative to the reference sample under the same developmental stages. Values are the mean ± standard deviation of three independent samples. *asterisk* indicates statistical significance (*p*<0.05), compared to the control.

Fig 2 describes the transcript levels of *pgm*, *ugp*, and *gls* genes in three stages of fruit body development of *G*. *lucidum* responding to different inductions. Ca^2+^ induction caused a significant improvement of the transcript of *ugp* gene in the three developmental stages, and the most enhancement was detected at the primordium stage, 2.63-fold that of the control. For both *pgm* and *gls* genes, they showed no response to this induction in the mycelium and primordium stages, while their expression was slightly up-regulated in the fruiting body stage, 1.32- and 1.22-fold that of their respective controls. SA treatment led to an increase in the expression of *pgm* and *ugp* in all three stages, in the range of 1.36–2.24, and 2.19–5.14 folds, respectively, in comparison with that of their respective controls. As to *gls* gene, SA induction had no any effect on its transcript in the mycelium and fruit body stages, but a slight up-regulation was observed in the primordium stage. After combined induction, the induced effect was similar to that observed for the SA induction for all three genes, but the most enhancing effect was detected for *ugp* gene in all three stages.

### Expression of six key genes involved in triterpenoid biosynthesis in response to different inductions

**Fig 3:**
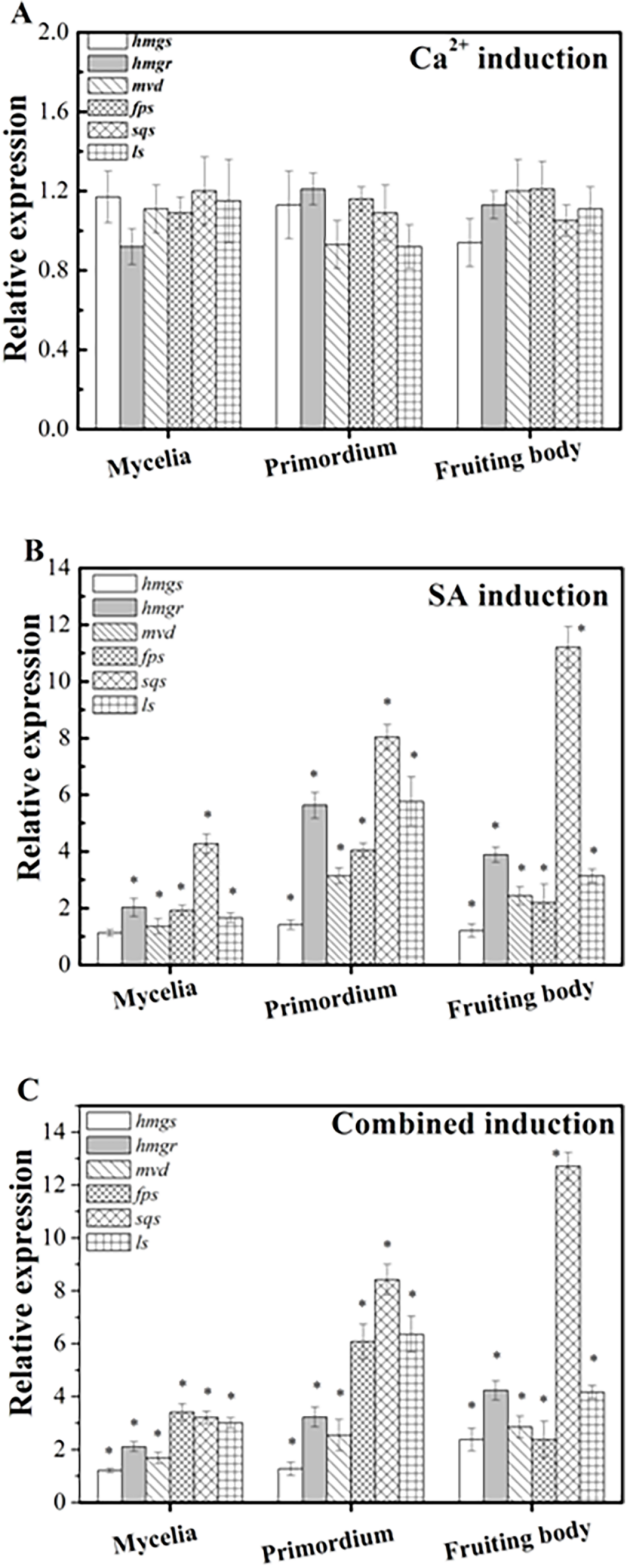
Transcript levels of the *hmgs*, *hmgr*, *mvd*, *fps*, *sqs*, and *ls* genes in the triterpenoid biosynthetic pathway during three stages of fruit body development of *G*. *lucidum* induced with Ca^2+^ and SA, alone or in combination. The measurement was carried out using samples after 3 days, 9 days, and 21 days of induction corrresponding the mycelium, primordium, and fruit body stages, respectively. Expression of the control samples in all three developmental stages is defined as 1.0, and expression levels of the induced samples are displayed as the ratio relative to the reference sample under the same developmental stages. Values are the mean ± standard deviation of three independent samples. *asterisk* indicates statistical significance (*p*<0.05), compared to the control.

Fig 3 shows the transcript levels of *hmgs*, *hmgr*, *mvd*, *fps*, *sqs*, and *ls* genes involved in triterpenoid biosynthesis in three stages of fruit body development of *G*. *lucidum* in response to different inductions. Using Ca^2+^ as the inducer, the expression of all six examined genes was almost unaffected in all three developmental stages, with no statistical difference (*p*>0.05). SA treatment up-regulated the mRNA levels of all examined genes with the exception of *hmgs* in the mycelium stage, and the most enhancement was observed for *sqs* gene (4.27-fold that of the control), and during the development of the mycelium into primordia, all examined genes were detected to be up-regulated, and were expressed at a higher level compared with the mycelium stage. Of six genes examined, *hmgr*, *sqs*, and *ls* positively responded to the SA induction most. In the fruit body stage, the transcript level of *sqs* gene became higher compared to that observed in the primordium, 11.21-fold that of the control, while the expression of the other genes was lower than those observed in the primordium stage. In the case of combined induction, the transcripts of six examined genes were up-regulated in the three developmental stages, but the extent of up-regulation differed considerably. A higher expression level was always observed during the primordium stage than during the mycelium stage for all examined genes, and when the primordium developed into fruit body, the expression of *hmgs* and *sqs* was further improved, but the other genes showed a decreasing trend, but still higher than those of their respective controls, with statistical difference at *p*<0.05.

## Discussion

Biologically active constituents in medicinal mushrooms have been of great interest since long. Dong et al. [41] reported that environmental conditions affected the accumulation of active components and the biological efficiency of *Cordyceps militaris*. Lin et al. [42] showed that the substrate not only affects the yield of *C*. *militaris*, but also the production of medicinally active components. Ultraviolet irradiation was found to be capable of improving the content of effective constituents, for example, by the conversion of ergosterol to D_2_ in harvested Oyster mushroom fruiting bodies [43]. Sudheer et al. [44] reported that ozone gas acts an inducer to enhance the level of bioactive compounds in harvested fresh *G*. *lucidum* fruiting bodies. However, data about the methods to be used to enhance the content of bioactive components in edible and medicinal mushroom fruiting bodies are still scarce.

To our knowledge, no published studies discuss available or new methods for the production of *G*. *lucidum* fruit bodies with high content of polysaccharides and triterpenoids during artificial cultivation. In the present study, the effect of induction by signal transduction in the fruiting stage on the production of active components in *G*. *lucidum* was examined for the first time. It was interestingly found that SA significantly improves the production of triterpenoids, but little effect was observed on polysaccharide production. Combining SA with calcium ion significantly increased the production of both polysaccharides and triterpenoids in the fruiting bodies, despite the fact that calcium ion had no positive effect on the production of polysaccharides and triterpenoids and that SA only strengthened triterpenoid production. Moreover, significant post-induction variation was observed in the content of several ganoderic acids. A similar response has also been reported under phenobarbital induction in the submerged culture of *G*. *lucidum* [20]. As a healthy and medical product, the improvement of polysaccharide and triterpenoid content in *G*. *lucidum* fruiting bodies would be important to the functional food and pharmacological industries.

In the present study, Ca^2+^ was found to increase the expression of *upg* gene in the mycelium, primordium, fruit body stages and that of *pgm*, and *gls* genes in fruit body stage, but no increase was noted in the polysaccharide content of the fruiting body. It was, therefore, suggested that the synthetic reactions catalyzed by the enzymes encoded by any of these genes are not rate-limiting steps in polysaccharide biosynthesis during fruit body development. This result is inconsistent with that observed for submerged cultures of *G*. *lucidum*, where improved content of polysaccharides by overexpression of *pgm* gene was obtained [27]. Further studies are warranted for more comprehensive understanding. Also, it has been reported that the addition of Ca^2+^ into submerged cultures of *G*. *lucidum* coupled with nitrogen limitation caused a significant improvement of ganoderic acid T content in the cultured mycelia [22]. The increased expression of *hmgr*, *sqs*, and *ls* genes has been suggested to be responsible for the enhanced production. In the present investigation, by employing Ca^2+^ on the mycelium, primordium, and fruiting body stages during fruit body development, the transcript of the six examined key genes in the triterpenoid biosynthetic pathway remained unchanged. This is possibly due to the uptake of sufficient amount of Ca^2+^ from the substrate by the mycelia.

SA in plants can alter various physiological process, such as photosynthesis, nitrogen metabolism, proline metabolism, the production of glycine betaine, and the antioxidant defense system [45,46]. A report done by Ren et al. [33] demonstrated that SA improved the expression level of *hmgs* gene, resulting in an improved ganoderic acid content in cultured mycelia. Cao et al. [34] reported that exogenous SA used in the submerged culture of *G*. *lucdium* significantly improved ganoderic acid content in the liquid-cultured mycelia, and found that the three key genes *hmgr*, *sqs*, and *osc* (*ls*) in the triterpenoid biosynthesis pathway were up-regulated by the induction. In the present study, spraying of SA onto the mycelium, primordium, and fruiting body during fruit body development significantly improved the content of triterpenoids in the fruiting body, and the transcript of *hmgs*, *hmgr*, *mvd*, *fps*, *sqs*, and *ls* genes was found to be significantly up-regulated. Increased production of triterpenoids may be attributed to the increased expression of these genes. This result is in line with that of previous studies [20,21], where the increased production of ganoderic acid coincided with enhanced expression of *hmgr*, *sqs*, and *ls* genes.

In the present study, the combined induction significantly improved the content of polysaccharides and triterpenoids in the fruiting bodies of *G*. *lucidum*. The enhanced transcript levels of *hmgs*, *hmgr*, *mvd*, *fps*, *sqs*, and *ls* genes may cause increased production of triterpenoids. Increase in the production of polysaccharides cannot be explained by analyzing the transcript levels of *pgm*, *upg*, and *gls* genes. It is possible that the transcript of one or more unidentified key genes involved in polysaccharide biosynthesis exerted a synergetic effect on its production because of the use of two inducing agents in combination. Further investigation is definitely warranted. To summarize, induction with signal transduction using a combination of SA and Ca^2+^ may be a good method for enhancing the production of polysaccharides and triterpenoids in the fruit bodies of *G*. *lucidum*, and holds great promise for the industrial production of polysaccharide- and triterpenoid-rich fruiting bodies.

## Supporting information

S1 Fig HPLC chromatograms of the extracts of the fruit bodies of *G*. *lucidum* obtained after different inductions. Sample symbols: SC, combined induction; S, SA induction; C, Ca^2+^ induction; E, ethanol treatment (control), EW, ethanol and distilled water treatment (control); W, distilled water treatment (control). Peak symbols: C2, ganoderic acid C2; C, ganoderic acid C; A, ganoderic acid A; LA, lucidenic acid A; E, ganoderic acid E; F, ganoderic acid F.
(TIF)
