# Supplementary figures and images for: Enhanced production of polysaccharides and triterpenoids in Ganoderma lucidum fruit bodies on induction with signal transduction during the fruiting stage
Source: PLoS One. 2018 Apr 25;13(4):e0196287. doi: 10.1371/journal.pone.0196287 (PMC5919040; doi:10.1371/journal.pone.0196287)

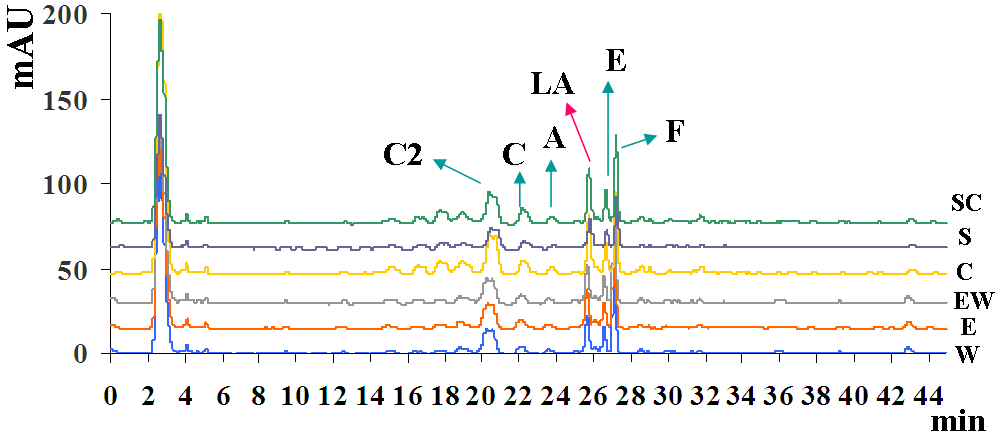

Supplement: S1 Fig — Sample symbols: SC, combined induction; S, SA induction; C, Ca2+ induction; E, ethanol treatment (control), EW, ethanol and distilled water treatment (control); W, distilled water treatment (control). Peak symbols: C2, ganoderic acid C2; C, ganoderic acid C; A, ganoderic acid A; LA, lucidenic acid A; E, ganoderic acid E; F, ganoderic acid F. (TIF) [file pone.0196287.s001.tif]
